# High-precision universality? A matched-pair sawbone study on the learning curve, accuracy, and implant interchangeability of a novel open-platform robotic UKA system

**DOI:** 10.3389/frobt.2026.1886514

**Published:** 2026-09-28

**Authors:** Pengyu Zhan, Ziyue Liu, Boran Liang, Yugang Xing, Xingyu Liu, Yiling Zhang, Jing Chen

**Affiliations:** 1 Peking University Aerospace Clinical Medical College, Beijing, China; 2 Aerospace Center Hospital, Department of Orthopedics, Beijing, China; 3 Beijing Changmugu Medical Technology Co., Ltd., Beijing, China

**Keywords:** implant switching, learning curve, open-platform system, robotic arthroplasty, sawbone model, unicompartmental knee arthroplasty

## Abstract

**Background:**

Robotic-assisted unicompartmental knee arthroplasty (UKA) improves implant positioning over conventional techniques, but commercial systems commonly bind the robotic platform to a single implant manufacturer, restricting clinical flexibility. Open-platform systems offer a vendor-neutral alternative, yet their effect on novice execution precision and workflow stability during implant switching remains insufficiently characterized.

**Methods:**

A prospective exploratory matched-pair case series was conducted on standardized synthetic bone models (Sawbones). Two robotic-naïve medical students, after identical training and baseline assessment, each performed 20 consecutive robotic UKA procedures on the ROPA open-platform system (Beijing Changmugu Medical Technology). Operator A used a single implant (Sigma HP Uni, DePuy Synthes); Operator B alternated between two manufacturer-specific workflows (DePuy Synthes; Kangernuo Medical). Because each condition included only one operator, observed differences reflect combined operator- and workflow-related factors that cannot be disentangled. Learning curves were assessed by Cumulative Sum (CUSUM). Three-dimensional resection accuracy was quantified by high-precision laser scanning. Overall perceived workload was assessed with NASA-TLX.

**Results:**

The precision CUSUM oscillated around zero for both operators (peak amplitudes +0.30 and −0.54 mm), indicating sub-millimeter accuracy from the first case without a detectable learning phase. All 40 cases fell within the ±1 mm safe zone (P > 0.05 across resection depths). Tibial valgus deviation was greater in Operator B (1.74° ± 1.33° vs. 0.97° ± 0.96°; P = 0.028, Wilcoxon signed-rank test), and the mean deviation of both operators remained below the ±2° degree threshold. Operator B reached the operative-time inflection point earlier (Case 5 vs. 10) and reported lower NASA-TLX workload (32.05 ± 25.60 vs. 38.35 ± 23.72, P = 0.010); this difference cannot be attributed to workflow alone.

**Conclusion:**

Under sawbone conditions—which cannot reproduce soft-tissue balancing, bleeding, anatomical variability, patient-specific deformity, or intraoperative decision-making—this two-operator exploratory evaluation found no evidence of compromised accuracy during alternating-implant workflows, with all 40 cases sub-millimeter. Findings are hypothesis-generating pre-clinical observations requiring validation in adequately powered, multi-operator prospective studies before clinical inference.

## 1 Introduction

Unicompartmental knee arthroplasty (UKA) is a technically demanding but functionally superior alternative to total knee replacement for isolated medial compartment osteoarthritis, offering faster recovery, greater preservation of proprioception, and improved functional outcomes (Motesharei et al., 2018; Harris et al., 2024; McEwen et al., 2024) when component positioning is accurate (Pearle et al., 2010; Kort et al., 2022). However, this sensitivity to technical precision represents a fundamental vulnerability of conventional UKA: even minor deviations in component alignment or resection depth are associated with accelerated polyethylene wear, component loosening, and clinical failure. Robotic-assisted surgical systems have emerged as a transformative technology to address this limitation, with multiple prospective studies and meta-analyses demonstrating improved implant positioning, reduced outlier rates, and greater reproducibility compared to conventional manual or navigation-guided techniques (Chin et al., 2021; Jiao et al., 2024; Fan et al., 2025).

Despite this promise, the prevailing commercial architecture for robotic arthroplasty systems often follows a “closed-platform” model (Chin et al., 2021; Fan et al., 2025), most notably the MAKO system (Stryker Corporation) (Vermue et al., 2020; Fan et al., 2025), in which the robotic platform is exclusively compatible with the implant portfolio of a single manufacturer (Chin et al., 2021; Kort et al., 2022). This proprietary design may create clinical and economic constraints (Moschetti et al., 2016). Hospitals that invest in a closed-platform system may become dependent on a single implant supplier, limiting competitive pricing advantages and the ability to leverage pre-existing implant inventory. Surgeons may also have less flexibility to select implants based on patient-specific anatomical considerations.

Open-platform robotic systems (Sun et al., 2024; Fan et al., 2025; He et al., 2025)—designed to operate with multiple manufacturers’ implants within a single hardware architecture—have been proposed as a clinically and economically rational alternative. However, a practical safety question remains: does frequent implant platform switching (Sun et al., 2024)—with its requirement to adapt surgical instruments and select implant-specific planning and execution parameters—compromise execution accuracy or increase overall perceived workload, particularly for operators early in their learning curve (Di Gialleonardo et al., 2025; Harato et al., 2025)? To date, prospective controlled evidence (Di Gialleonardo et al., 2025; Rizvi et al., 2025) remains limited under conditions of multi-manufacturer implant alternation.

The present study was designed to address this evidence gap. Using standardized synthetic bone models (Sawbones) (Iñiguez et al., 2021), we conducted a prospective exploratory matched-pair case series to characterize learning curve dynamics, three-dimensional execution accuracy, and workflow performance during implant switching (Negrín et al., 2021; Gaggiotti et al., 2024; Di Gialleonardo et al., 2025) in a novel open-platform robotic UKA system. We proposed two *a priori* hypotheses for exploratory evaluation: (1) that the system’s error-threshold safety interlock would be associated with sub-millimeter execution accuracy across two implant-specific workflows and early operator experience; and (2) that multi-implant alternation would not be associated with substantially higher overall perceived workload relative to single-platform operation. Because each workflow condition was represented by a single operator, these hypotheses are evaluated in a descriptive and hypothesis-generating framework rather than a confirmatory one.

## 2 Materials and methods

### 2.1 Study design and participants

This study employed a prospective exploratory matched-pair case series design (Iñiguez et al., 2021; Negrín et al., 2021) using standardized synthetic bone models (Sawbones) to characterize learning curve dynamics, three-dimensional execution accuracy, and workflow performance during implant switching in a novel open-platform robotic system for Unicompartmental Knee Arthroplasty (UKA). Because each workflow condition was represented by a single operator, the study does not constitute a controlled group-level experiment; all findings should be interpreted as descriptive and hypothesis-generating, with observed inter-operator differences reflecting a combination of individual operator characteristics and workflow-related factors that cannot be disentangled in the present design.

Two senior medical students with comparable academic backgrounds but no prior experience in robotic surgery were recruited as participants. Both participants underwent an identical standardized training curriculum, which included theoretical learning (2 h), dry-lab simulation (2 h), and observation of robotic surgeries (5 cases). Prior to the formal experiment, both participants were required to pass a strict ‘Baseline Assessment’, mandating the independent, zero-error completion of a full system registration and bone resection workflow in a dry-lab setting.

Operator A (single-implant workflow): Performed 20 consecutive robotic-assisted UKA procedures using a single implant system (Sigma HP Uni, DePuy Synthes).

Operator B (alternating-implant workflow): Performed 20 consecutive procedures using a mixed-implant protocol, alternating between Implant A (DePuy Synthes) and Implant D (Kangernuo Medical) to simulate a multi-manufacturer implant-switching workflow.

To strictly eliminate chronological observation bias, an opaque physical barrier was established between the two operators during Operator A’s 20 cases, ensuring Operator B commenced with an identical baseline of zero physical robotic execution experience.

### 2.2 Robotic system and bone models

All procedures were performed using the ROPA robotic system (Beijing Changmugu Medical Technology Co., Ltd., Beijing, China) (Jones et al., 2019; Sun et al., 2024; He et al., 2025), comprising an optical navigator, a robotic arm vehicle, and a main control trolley. The optical navigator tracks reflective rigid-body tracers attached to the femur and tibia, providing real-time spatial feedback. The system continuously monitors tool position relative to the planned resection plane; when deviation exceeds the pre-defined safety threshold, an automatic pause is triggered to prevent excessive osteotomy or soft tissue damage. The two implant systems evaluated—DePuy Synthes Sigma HP Uni and Kangernuo Medical Fixed-Bearing UKA—share key dimensional equivalences relevant to a controlled implant-switching comparison: identical distal femoral resection depth (6.7 mm), identical tibial tray base thickness (2 mm), and an identical 6-size tibial tray AP/ML dimension matrix (Table 1), independent of their differing posterior condyle cutting strategies and fixation geometries. Standardized synthetic tibia and femur models, manufactured from a uniform single-patient CT dataset, were utilized for all 40 procedures to minimize anatomy-related confounding variables. The geometric and operational characteristics relevant to the implant-switching workflow are detailed in Table 1.

**TABLE 1:** Implant-related characteristics relevant to robotic planning and switching workflow.

Technical parameter	Implant A (DePuy Synthes/J&J)	Implant D (Kangernuo Medical)	Potential risk if parameters are confused
Femoral Articular Curvature (surface geometry)	Multi-radius design; larger posterior condyle radius. Full articular contact maintained at 155° flexion. 7° internal rotation built into asymmetric component. Coronal transition edge permits ≤10° off-parallel articulation^a^	Multi-radius design; full articular contact at 155° flexion. 7° internal rotation. Asymmetric left/right design. Specific sagittal curvature radii proprietary^a^	Potential mismatch in flexion/extension gap balancing
Standard Resection Depth (mm) (distal/posterior condyle)	Distal: 6.7 mm (constant across all 6 sizes). Posterior: 105° cutting angle (TRI-ONE block). Optional +1.5 mm additional posterior resection. Constant peg-to-cut distance permits up/downsizing after trialing^a^	Distal: 6.7 mm (constant). Posterior: 15.3 mm. Optional +1.5 mm additional posterior resection. Equal-thickness posterior condyle design^a^	Potential over-resection or under-resection of bone stock
Tibial Tray Base Thickness (mm)	2 mm (metal tray). Total construct 7–11 mm with insert. 6 sizes (AP 41.5–56.5 mm; ML 24–34 mm). Anterior tip radiused to 4.5 mm. Cobalt-chrome, high-polish^a^	2 mm (metal tray). Total construct 7–11 mm with insert. 6 sizes (AP 41.5–56.5 mm; ML 24–34 mm)^b^. Cobalt-chrome, high-polish^a^	Potential joint line elevation or depression, affecting kinematics
Tibial Fixation Design (keel/peg geometry)	Femoral: two parallel pegs with undercuts and anti-pressurization flutes. Tibial: tripod keel-and-peg design. Wider keel enhances rigidity. All cemented^a^	Femoral: two parallel posts with grooves. Tibial: dual parallel posts with triangular stabilization pattern. Bone-preserving slotted keel. All cemented^a^	Potential milling of incorrect slot geometry, compromising tibial fixation

^a^ Both systems employ multi-radius femoral geometries with proprietary sagittal curvature values. The identical distal resection depth (6.7 mm), comparable gap-balancing protocols, and identical tibial tray size matrix (AP, 41.5–56.5 mm x ML, 24–34 mm across 6 sizes) indicate broad comparability in the parameters most directly related to bone preparation. Data sourced from the DePuy Synthes Sigma HP, Partial Knee Technical Monograph and the Kangernuo Medical Fixed-Bearing UKA, Product Introduction (provided by respective manufacturers).

^b^ The two tibial trays share an identical 6-size AP/ML, dimension matrix, supporting a controlled comparison within similar bone-preparation constraints.

Two size-2 UKA prostheses from different manufacturers were used to simulate implant switching during robotic-assisted bone preparation. Because the overall geometry of the two prostheses was broadly comparable, the comparison focused on implant-specific planning and workflow parameters rather than on substantial morphologic differences between implants. Implant-related characteristics relevant to robotic planning and intraoperative switching are summarized in Table 1.

### 2.3 Surgical workflow

The surgical workflow was standardized into five time-interval phases applicable to both implant systems. Phase one (P1: System Setup) encompassed robotic arm initialization, optical navigator calibration, and implant-specific parameter loading into the planning software; P1 was excluded from Total Operative Time as it does not involve patient contact. Phase 2 (P2: Pre-operative Preparation) covered positioning frame installation and registration pin insertion into the femur and tibia. Phase 3 (P3: Registration and Verification) covered optical surface registration of anatomical landmarks, automated verification of tracker rigidity, and surgeon approval of the pre-operative plan; this phase contains the primary implant-switching adaptation point, as the operator selects the implant-specific bone-cutting parameters (resection depth, cutting angle) within the software. Phase 4 (P4: Resection and Trialing) covered robotic bone cutting under continuous position monitoring and trial implant installation; the system’s error-threshold safety interlock automatically pauses the robotic arm if tool deviation exceeds the pre-defined threshold. Phase 5 (P5: Final Verification) covered alignment and range-of-motion assessment (Kwon et al., 2019). Steps in P2 and P4 were identical across both implant workflows. Steps in P3 differed in that Operator B was required to re-select implant-specific parameters at the software interface when alternating between Implant A and Implant D. Total Operative Time (OT) = P2 + P3 + P4 + P5.

### 2.4 Data collection and statistical analysis

#### 2.4.1 Accuracy assessment

Post-operative measurements were conducted using a high-precision 3D laser scanner (Jones et al., 2019; Gaggiotti et al., 2024) to obtain surface STL files of the resected bones. Actual resection planes and alignment angles were reconstructed and compared with pre-operative plans. The Mean Absolute Resection Error (MARE) was calculated per case by averaging the absolute depth deviations of the distal femur, posterior condyle, and proximal tibia. Key parameters included: (1) Bone Resection Thickness (mm): distal femur, posterior condyle, femoral condyle, proximal tibia, and tibial plateau; (2) Component Alignment (°): femoral/tibial valgus angle and Posterior Tibial Slope (PTS); (3) Overall Limb Alignment (°): HKA, JLCA, LDFA, MPTA, and aHKA (see Figure 1 for anatomical definitions). Psychological workload was assessed immediately after each case using the NASA-TLX.

**FIGURE 1:**
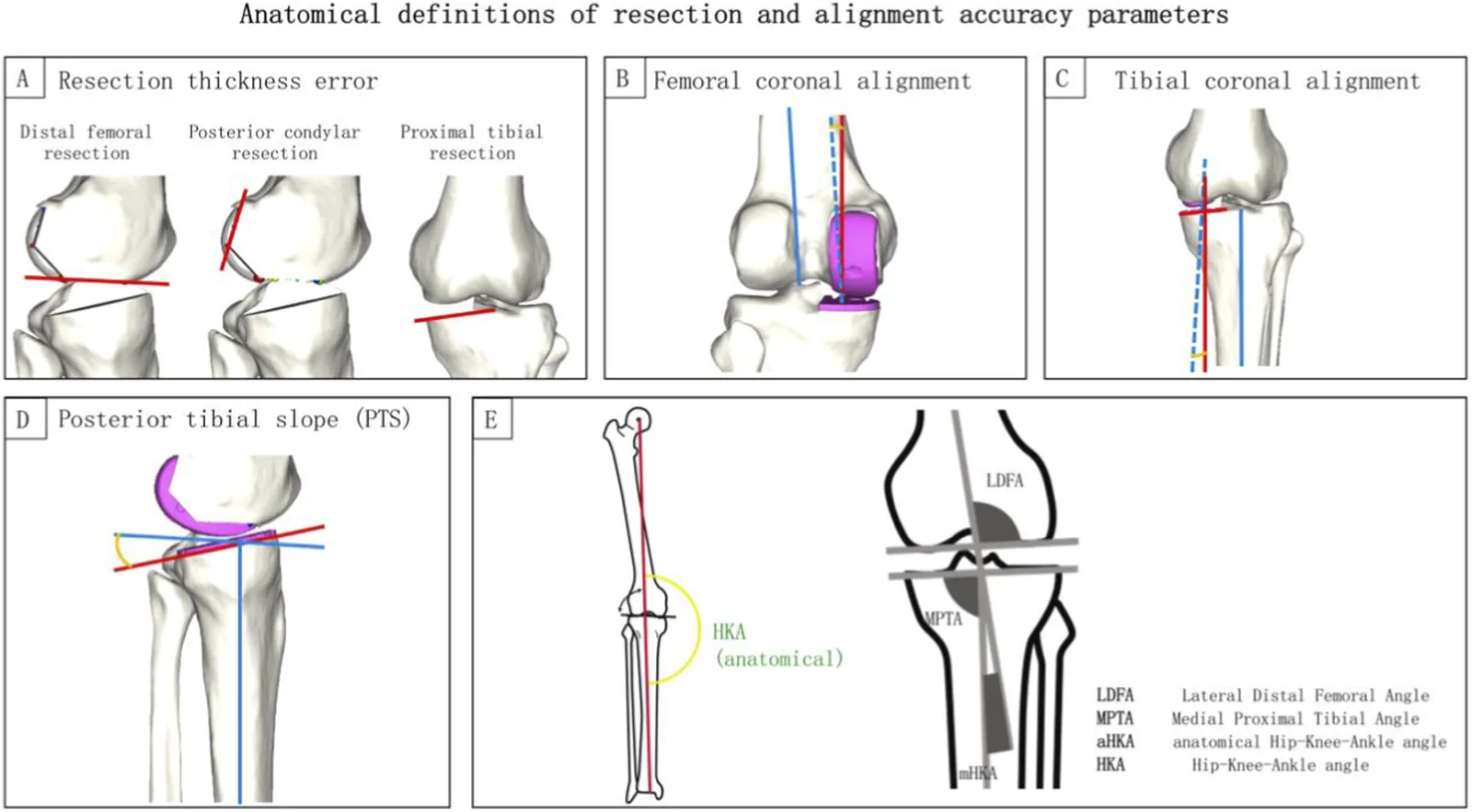
Anatomical definitions of the resection-thickness and alignment-accuracy parameters assessed in this study. **(A)** Resection-thickness error at the three principal resection planes—distal femoral, posterior femoral-condylar, and proximal tibial—quantified as the perpendicular offset between the planned (yellow/green markers) and the actual (red markers) resection surfaces, with the signed difference Δt = actual − planned; positive Δt denotes over-resection and negative Δt denotes under-resection. **(B)** Femoral coronal alignment, quantified by the Lateral Distal Femoral Angle (LDFA), defined as the lateral angle formed between the femoral anatomical (diaphyseal) axis (solid blue line) and the distal femoral joint-line tangent (solid red line); the mechanical axis reference is shown as a dashed blue line, and the LDFA arc is drawn in orange at the distal-lateral femoral condyle. **(C)** Tibial coronal alignment, quantified by the Medial Proximal Tibial Angle (MPTA), defined as the medial angle formed between the tibial anatomical axis (solid blue line) and the proximal tibial joint-line tangent (solid red line); the mechanical axis reference is shown as a dashed blue line, and the MPTA arc is drawn in orange at the medial proximal tibia. **(D)** Posterior Tibial Slope (PTS), measured in the sagittal plane as the angle between the tibial-plateau tangent (red) and the perpendicular to the tibial long axis (blue), highlighted by the orange arc. **(E)** Whole-limb frontal-plane schematic providing a global-alignment cross-reference for panels B–D. The left inset shows the anatomical hip-knee-ankle angle (aHKA, yellow arc) referenced to the femoral and tibial anatomical (diaphyseal) axes (red). The right inset shows a radiographic-style whole-limb outline in which the mechanical hip-knee-ankle angle (mHKA) is drawn along the femoral-head-to-ankle-centre mechanical axis (grey), together with the LDFA and MPTA arcs indicated in their anatomical locations, thereby linking the local views in panels B–C to the global limb-alignment framework used throughout the Results. Abbreviations: LDFA, Lateral Distal Femoral Angle; MPTA, Medial Proximal Tibial Angle; aHKA, anatomical Hip-Knee-Ankle angle; mHKA, mechanical Hip-Knee-Ankle angle; PTS, Posterior Tibial Slope; Δt, resection-thickness error (actual − planned). All angular and linear definitions follow the radiographic conventions cited in the Methods section.

#### 2.4.2 Statistical analysis

Analyses were performed in GraphPad Prism v9.0 (GraphPad Software, San Diego, CA, United States of America). Measurement error = |Valuepost − Valueplan|. Normality of paired differences was assessed by the Shapiro-Wilk test. Where paired differences were normally distributed, the Paired Student's t-test was applied; otherwise, the Wilcoxon matched-pairs signed-rank test was used. Each case performed by Operator A was paired with the corresponding case by sequential case number performed by Operator B (Case 1 with Case 1, etc.), under the rationale that both operators began with identical baseline training and were exposed to equivalent cumulative experience at each case number. Because the study involves two individual operators rather than independent sample groups, all reported P-values are descriptive indicators of inter-operator variation and should not be interpreted as evidence of condition-level effects. Overall perceived workload was assessed immediately after each case using the Raw TLX total score of the NASA Task Load Index (NASA-TLX), a multidimensional instrument encompassing six subscales: mental demand, physical demand, temporal demand, performance, effort, and frustration. Learning curve data were analyzed by CUSUM method with second-order polynomial regression. Safe zones were defined as ±1 mm (resection depth) and ±2° (alignment); outlier rates were compared by Fisher’s exact test. P < 0.05 was considered statistically significant for descriptive purposes. To mitigate potential commercial bias arising from the affiliation of two authors (X.L. and Y.Z.) with the robotic system manufacturer, the following measures were implemented: (1) all laser scanning and post-operative three-dimensional measurements were performed by authors without manufacturer affiliation; (2) statistical analyses were conducted independently of the manufacturer-affiliated authors; and (3) the manufacturer-affiliated authors were involved solely in technical system configuration and implant parameter verification, and were not involved in data collection, post-operative measurement, or statistical analysis.

## 3 Results

### 3.1 Learning curve analysis

CUSUM analysis revealed a marked divergence between operative efficiency and execution accuracy (Figure 2) (Di Gialleonardo et al., 2025; Harato et al., 2025). For operative time, Operator B (alternating-implant workflow) reached the predefined operative-time inflection point by Case 5, while Operator A (single-implant workflow) reached a comparable point by Case 10. In contrast, the precision CUSUM based on Mean Absolute Resection Error (MARE) displayed sub-millimeter accuracy from the first case for both operators, with no detectable initial period of lower precision: both oscillated tightly around the zero-axis in a random-walk pattern, with maximum peak amplitudes of +0.30 mm and −0.54 mm. Resection accuracy was comparable between the first and 20th case for both operators.

**FIGURE 2:**
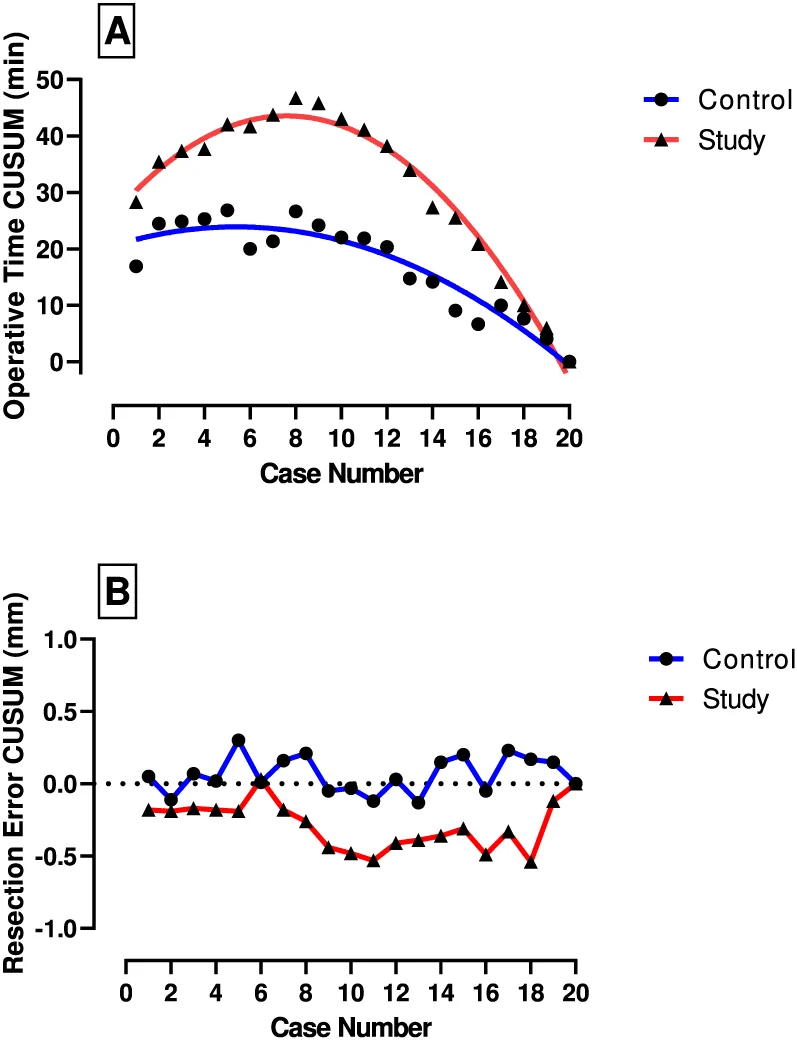
Cumulative Sum (CUSUM) analysis of **(A)** operative time and **(B)** execution accuracy. Operator B (alternating-implant, red) reached the predefined operative-time inflection point at Case 5; Operator A (single-implant, blue) at Case 10. The precision CUSUM values (panel **B**) are CUSUM-derived, based on Mean Absolute Resection Error (MARE), oscillating around zero with maximum amplitudes of +0.30 mm and –0.54 mm, suggesting no discernible accuracy-learning phase under the current sawbone conditions.

### 3.2 Accuracy verification and safety thresholds

Sub-millimeter accuracy was consistently achieved across all three primary resection planes (Figures 2–5) (Pearle et al., 2010; Park et al., 2019; Gaggiotti et al., 2024). The mean absolute errors for the distal femur were 0.54 ± 0.32 mm (Control) vs. 0.41 ± 0.29 mm (Study) (P = 0.19); for the posterior condyle, 0.49 ± 0.28 mm vs. 0.57 ± 0.31 mm (P = 0.36); and for the proximal tibia, 0.43 ± 0.23 mm vs. 0.34 ± 0.20 mm (P = 0.22). No significant inter-group differences were detected for any resection plane (all P > 0.05). All 40 cases remained within the predefined ±1 mm safe zone (Fisher’s exact test: P > 0.99 between groups).

**FIGURE 3:**
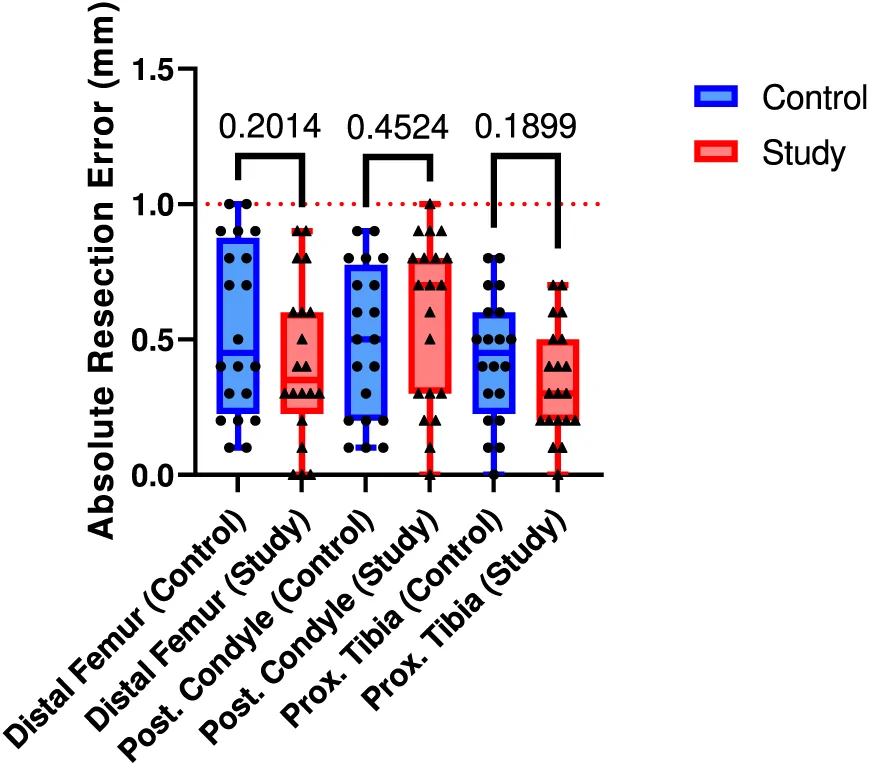
Resection accuracy of primary bone surfaces. Box-and-whisker plots comparing absolute resection errors (mm) for distal femur, posterior condyle, and proximal tibia between Operator A (single-implant, blue) and Operator B (alternating-implant, red). Dotted line denotes the ±1 mm safe zone (safety threshold). All plotted values are absolute errors in millimeters. No significant differences between operators were observed for any resection plane (all P > 0.05).

**FIGURE 4:**
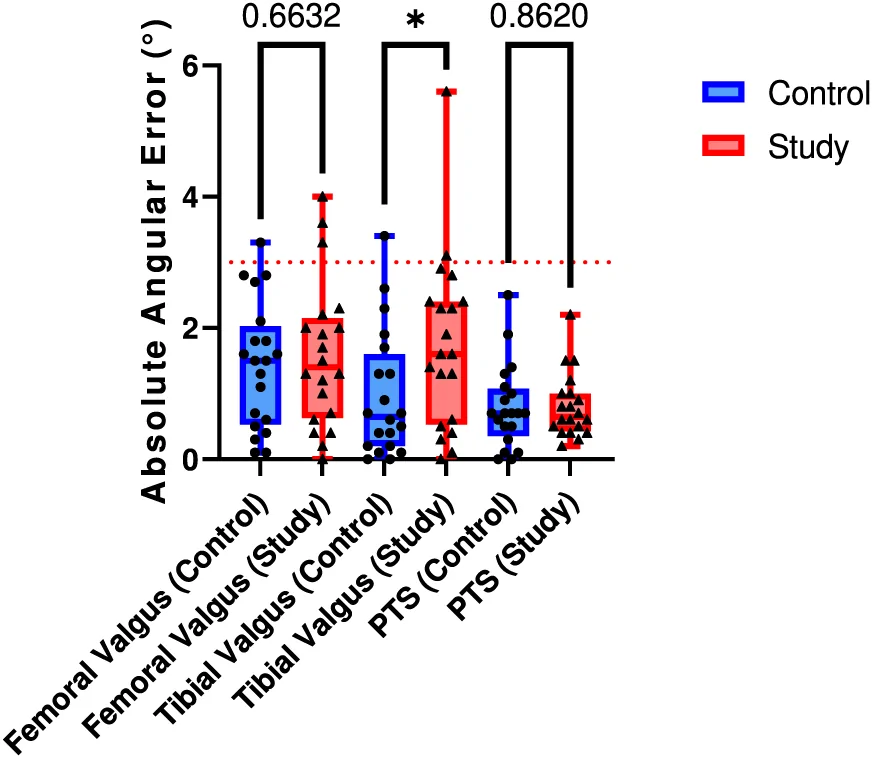
Component alignment deviations. Comparison of femoral valgus angle, tibial valgus angle, and Posterior Tibial Slope (PTS) deviations between Operator A (single-implant, blue) and Operator B (alternating-implant, red). Dotted line indicates the ±2° safety threshold. Tibial valgus deviation was significantly greater in Operator B (P = 0.028 by Wilcoxon matched-pairs signed-rank test; asterisk indicates P < 0.05). Femoral valgus and PTS did not differ significantly (P > 0.05). All values represent absolute errors (degrees); the mean deviation of every parameter in both operators remained below the threshold.

**FIGURE 5:**
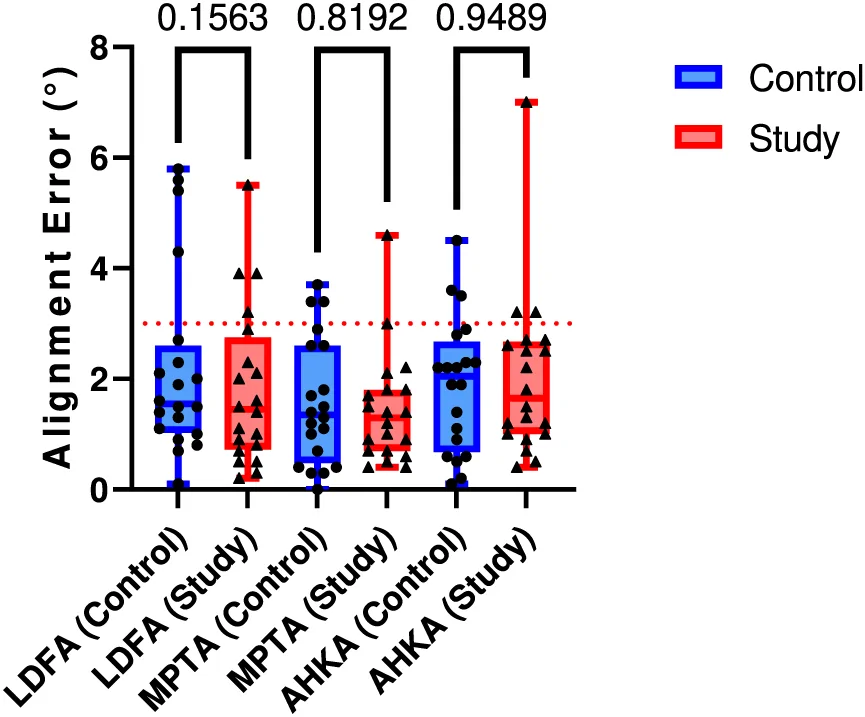
Overall limb alignment accuracy. Absolute deviations in LDFA, MPTA, and aHKA between Operator A (single-implant, blue) and Operator B (alternating-implant, red). One outlier (aHKA deviation of 7.0 degrees in Operator B, Case 5) was attributable to a ligament simulation failure unrelated to robotic execution.

Among component alignment deviations, femoral valgus (1.43° ± 0.97° vs. 1.58° ± 1.12°, P = 0.65) and posterior tibial slope (0.79° ± 0.63° vs. 0.81° ± 0.49°, P = 0.91) did not differ significantly between operators. Tibial valgus deviation was statistically significantly greater in Operator B than Operator A (1.74° ± 1.33° vs. 0.97° ± 0.96°; P = 0.028 by Wilcoxon matched-pairs signed-rank test; Figure 4). Notably, the mean deviation of both operators remained below the predefined ±2° degree safety threshold; the difference is therefore of statistical but not necessarily clinical significance under the current experimental conditions. Full-limb alignment parameters were comparable: LDFA (2.20° ± 1.71° vs. 1.81° ± 1.43°, P = 0.44), MPTA (1.58° ± 1.15° vs. 1.44° ± 1.01°, P = 0.67), and aHKA (1.89° ± 1.21° vs. 2.00° ± 1.48°, P = 0.78) (Figure 5) (Alruwaili et al., 2022; Harris et al., 2024). One outlier (aHKA deviation of 7.0° in Operator B, Case 5) was attributable to an intraoperative ligament simulation failure unrelated to robotic execution. Sensitivity analysis confirmed that inclusion of this outlier did not alter the primary conclusions: aHKA remained non-significantly different between operators both with the outlier included (1.89° ± 1.21° vs. 2.00° ± 1.48°, P = 0.79) and with it excluded (1.93° ± 1.23° vs. 1.74° ± 0.92°, P = 0.60).

Simple linear regression of absolute aHKA alignment error against case number showed no significant trend toward improvement or deterioration in either group (P > 0.05 for both; Figure 6), supporting the absence of a detectable accuracy-learning trend (Park et al., 2019; Di Gialleonardo et al., 2025).

**FIGURE 6:**
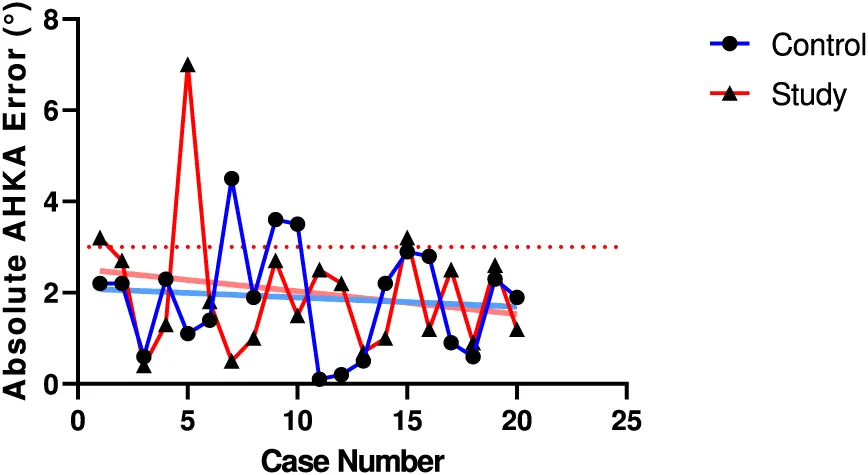
Simple linear regression of absolute anatomical hip-knee-ankle (aHKA) alignment error over consecutive cases. Fitted regression slopes for both groups were not significantly different from zero (P > 0.05), supporting stable accuracy throughout the learning period and the absence of a detectable accuracy-learning trend.

### 3.3 Workload assessment

Operator B (alternating-implant workflow) reported significantly lower overall NASA-TLX workload scores compared to Operator A (single-implant workflow) (32.05 ± 25.60 vs. 38.35 ± 23.72; P = 0.010 by paired t-test; Figure 7). The large standard deviations in both operators reflect substantial within-operator variability across the 20-case series, consistent with a learning process. It should be noted that this difference in overall perceived workload cannot be attributed to the implant-switching workflow condition alone; as each condition involved only one operator, individual differences between operators represent an equally plausible explanation. The finding that the alternating-implant operator did not report higher overall workload than the single-implant operator provides preliminary evidence against a workload-increase hypothesis, but does not confirm equivalence.

**FIGURE 7:**
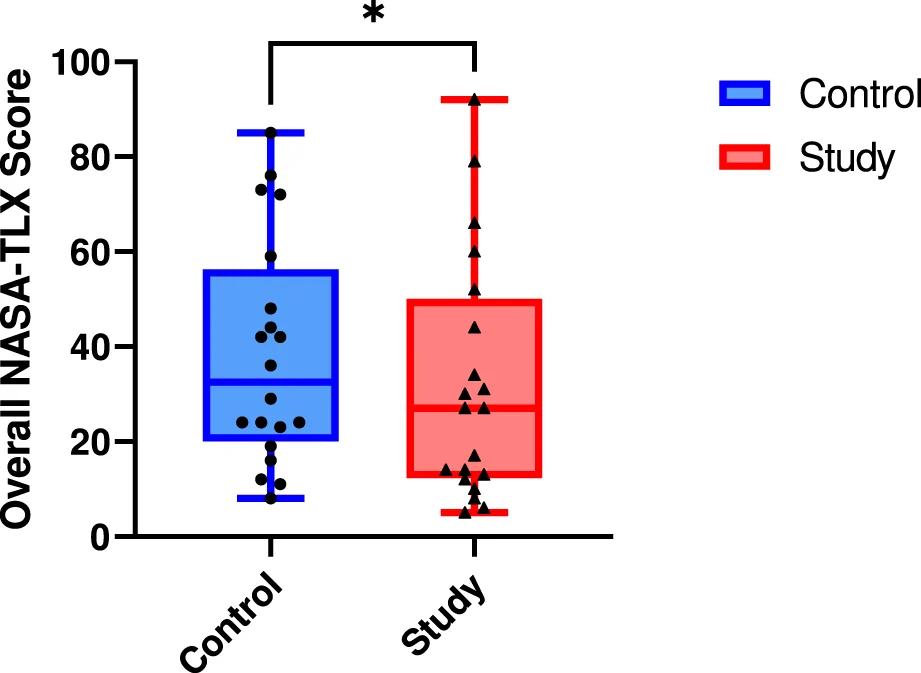
NASA Task Load Index (NASA-TLX) overall perceived workload comparison. Operator B (alternating-implant, red) reported lower Raw TLX total scores than Operator A (single-implant, blue): 32.05 ± 25.60 vs. 38.35 ± 23.72 (P = 0.010 by paired t-test). The large standard deviations reflect substantial within-operator variability. This difference cannot be attributed to the workflow condition alone, as each condition was represented by a single operator.

## 4 Discussion

### 4.1 Absence of a detectable accuracy-learning phase under sawbone conditions

The main finding of this study is the divergence between the learning curves for operative efficiency and execution accuracy. While the operative time CUSUM exhibited the expected sigmoid pattern with clear inflection points—consistent with classical motor skill acquisition theory (Di Gialleonardo et al., 2025)—the precision CUSUM demonstrated no detectable accuracy-learning phase: accuracy remained sub-millimeter and stable from the first case, despite subsequent gains in operative speed.

This absence of a detectable accuracy-learning phase may be partly explained by the error-threshold safety interlock mechanism (Vermue et al., 2020; Fan et al., 2025) inherent in modern open-platform robotic systems. Unlike conventional manual instrumentation, the robotic system continuously monitors tool position relative to the planned resection plane; when deviation exceeds a pre-defined threshold, the system triggers an automatic pause and alerts the surgeon (Vermue et al., 2020; Fan et al., 2025), helping maintain cuts within a three-dimensional safety envelope. The surgeon’s role shifts from direct precision execution toward verification and supervised execution, reducing the trial-and-error component that traditionally characterizes surgical skill acquisition. Under the present sawbone conditions, our data suggest that novice operators can achieve comparable sub-millimeter resection accuracy from their first robotic case to their 20th. The safety net provided by this real-time error-threshold feedback loop may lower the barrier to achieving high-precision robotic bone preparation.

### 4.2 Performance during cross-platform practice

Operator B (alternating-implant workflow) reached the predefined operative-time inflection point earlier (Case 5 vs. Case 10) and reported lower overall perceived workload (P = 0.010) compared to Operator A (single-implant workflow). We offer a ‘cross-platform schema construction’ hypothesis (Di Gialleonardo et al., 2025; Harato et al., 2025) as one possible but hypothesis-generating interpretation. When operating with a single implant platform, the operator may develop platform-specific procedural memory focused on repeated step execution. In contrast, alternating between two implant workflows may encourage the operator to extract underlying structural principles—spatial registration logic, feedback interpretation, and response calibration—rather than memorizing a fixed sequence. This may accelerate the formation of transferable motor schemas and reduce perceived workload, consistent with established findings in motor learning research that variable practice can improve retention and transfer compared to blocked practice. However, it is equally possible that the lower workload reported by Operator B reflects individual differences in baseline cognitive aptitude or prior experience rather than a workflow effect. These two explanations cannot be distinguished in the present two-operator design.

### 4.3 Implant switching within an open-platform workflow

In this exploratory two-operator evaluation, alternating-implant workflow was not associated with compromised resection accuracy or increased overall perceived workload under the present standardized sawbone conditions. Operator B, alternating between two manufacturer-specific implant workflows, achieved comparable or better performance on most outcomes relative to Operator A. Notably, tibial valgus deviation was significantly greater in Operator B (P = 0.028), though the mean deviation of both operators remained below the ±2° degree safety threshold; this finding may reflect individual anatomical measurement variability or minor differences in registration technique rather than a workflow-related effect. Post-hoc engineering analysis confirmed that the two implant systems share identical tibial tray base thickness (2 mm) and an identical 6-size AP/ML dimension matrix (Table 1). This dimensional convergence reduces the need for differential bone preparation when switching between the two systems and provides a mechanistic basis for the observed precision equivalence in this experiment; findings should therefore not be generalized to implant systems with substantially different dimensional specifications. In conventional manual surgery, frequent switching between implant systems may introduce risks of instrument confusion and parameter-setting errors. The open-platform robotic system may mitigate these risks through software-enforced error-proofing and the error-threshold safety interlock (Vermue et al., 2020; Fan et al., 2025), reducing the likelihood that workflow switching translates into resection error. These data support the feasibility of further evaluating open-platform robotic workflows as a means of expanding clinical flexibility, while clearly requiring validation in larger, properly controlled studies (Chin et al., 2021; Kort et al., 2022; Rizvi et al., 2025).

### 4.4 Limitations

Several limitations warrant acknowledgment. Most critically, this study involved only two operators, one per workflow condition. This means that observed inter-operator differences reflect an inseparable combination of individual operator characteristics and workflow-related effects; no causal attribution to the implant-switching protocol can be made. The study should be regarded as an exploratory pre-clinical feasibility evaluation, not as a controlled experiment with inferential group-level conclusions. The findings should be validated in adequately powered, multi-operator prospective studies (Kort et al., 2022; Di Gialleonardo et al., 2025). Second, the sawbone model cannot replicate soft-tissue balancing, intraoperative bleeding, patient-specific anatomical deformity, or complex intraoperative decision-making (Iñiguez et al., 2021; Negrín et al., 2021); the model was intentionally selected as a practical and ethically appropriate medium for a controlled multi-platform benchmarking experiment. Third, chronological confounding cannot be entirely excluded, though the direction of this bias would favor Operator A, making our findings conservative. Fourth, the two prosthesis systems share several dimensional parameters; findings should not be interpreted as evidence of universal interchangeability among substantially different implant designs. Fifth, overall perceived workload was assessed by the NASA-TLX total score, which aggregates six heterogeneous subscales; future studies should examine individual subscale responses and incorporate objective workload metrics such as heart rate variability or pupillometry. Finally, generalizability to total knee arthroplasty, clinical UKA, or other robotic procedures requires separate investigation.

## 5 Conclusion

This prospective exploratory case series found that, under standardized sawbone conditions—which cannot reproduce soft-tissue complexity, intraoperative bleeding, patient-specific anatomical deformity, or complex clinical decision-making—a novel open-platform robotic UKA system maintained sub-millimeter resection accuracy for both a single-implant and an alternating-implant workflow from the first case onward (Sun et al., 2024; He et al., 2025). Three preliminary observations emerge: first, no detectable accuracy-learning phase was observed in either operator, with precision remaining stable across all 20 cases (Park et al., 2019; Gaggiotti et al., 2024; Di Gialleonardo et al., 2025); second, the alternating-implant operator did not demonstrate reduced accuracy or higher overall perceived workload than the single-implant operator, providing preliminary evidence against a workflow-compromise hypothesis; and third, the system’s error-proofing mechanisms may contribute to maintaining precision during multi-manufacturer implant workflows. These observations are hypothesis-generating and should not be interpreted as evidence of clinical superiority or universal implant interchangeability. Validation in adequately powered, multi-operator, and ultimately clinical studies is required before the open-platform paradigm can be recommended for routine clinical adoption (Chin et al., 2021; Fan et al., 2025; Rizvi et al., 2025).

## Data Availability

The original contributions presented in the study are included in the article/Supplementary Material, further inquiries can be directed to the corresponding author.
