# Supplementary material for: High-precision universality? A matched-pair sawbone study on the learning curve, accuracy, and implant interchangeability of a novel open-platform robotic UKA system
Source: Front Robot AI. 2026 Sep 28;13:1886514. doi: 10.3389/frobt.2026.1886514 (PMC13654760; doi:10.3389/frobt.2026.1886514)
Supplement: Supplementary file 1 [file Table2.docx]

Supplementary Material

# 1 Supplementary Data

**Supplementary Data Sheet 1.** Cleaned public dataset for operative time and CUSUM analysis, three-dimensional resection accuracy and CUSUM analysis, component alignment, limb-alignment deviation and CUSUM analysis, and NASA-TLX workload assessment across all 40 robotic UKA procedures. The workbook contains eight sheets: README, Data_Dictionary, Case_Info, Operative_Time_CUSUM, Resection_Accuracy_CUSUM, Component_Alignment, Limb_Alignment_CUSUM, and NASA_TLX. Unused internal working columns not analyzed in the manuscript were omitted from the public workbook; the original source workbook is retained by the authors. Operator identities are anonymized as Surgeon A (Control group; single-implant workflow) and Surgeon B (Study group; mixed-implant workflow).

No supplementary figures or supplementary tables are included in this file. The main figures are submitted separately as figure files according to Frontiers requirements.
